# Alginate-Based Micro- and Nanosystems for Targeted Cancer Therapy

**DOI:** 10.3390/md20100598

**Published:** 2022-09-23

**Authors:** Siavash Iravani, Rajender S. Varma

**Affiliations:** 1 Faculty of Pharmacy and Pharmaceutical Sciences, Isfahan University of Medical Sciences, Isfahan 81746-73461, Iran; 2 Regional Centre of Advanced Technologies and Materials, Czech Advanced Technology and Research Institute, Palacký University in Olomouc, Šlechtitelů 27, 783 71 Olomouc, Czech Republic

**Keywords:** alginate, alginate-based nanosystems, nanomaterials, cancer therapy, drug delivery

## Abstract

Alginates have been widely explored due to their salient advantages of hydrophilicity, biocompatibility, mucoadhesive features, bioavailability, environmentally-benign properties, and cost-effectiveness. They are applied for designing micro- and nanosystems for controlled and targeted drug delivery and cancer therapy as alginate biopolymers find usage in encapsulating anticancer drugs to improve their bioavailability, sustained release, pharmacokinetics, and bio-clearance. Notably, these nanomaterials can be applied for photothermal, photodynamic, and chemodynamic therapy of cancers/tumors. Future explorations ought to be conducted to find novel alginate-based (nano)systems for targeted cancer therapy using advanced drug delivery techniques with benefits of non-invasiveness, patient compliance, and convenience of drug administration. Thus, some critical parameters such as mucosal permeability, stability in the gastrointestinal tract environment, and drug solubility ought to be considered. In addition, the comprehensive clinical translational studies along with the optimization of synthesis techniques still need to be addressed. Herein, we present an overview of the current state of knowledge and recent developments pertaining to the applications of alginate-based micro- and nanosystems for targeted cancer therapy based on controlled drug delivery, photothermal therapy, and chemodynamic/photodynamic therapy approaches, focusing on important challenges and future directions.

## 1. Introduction

**Figure 1:**
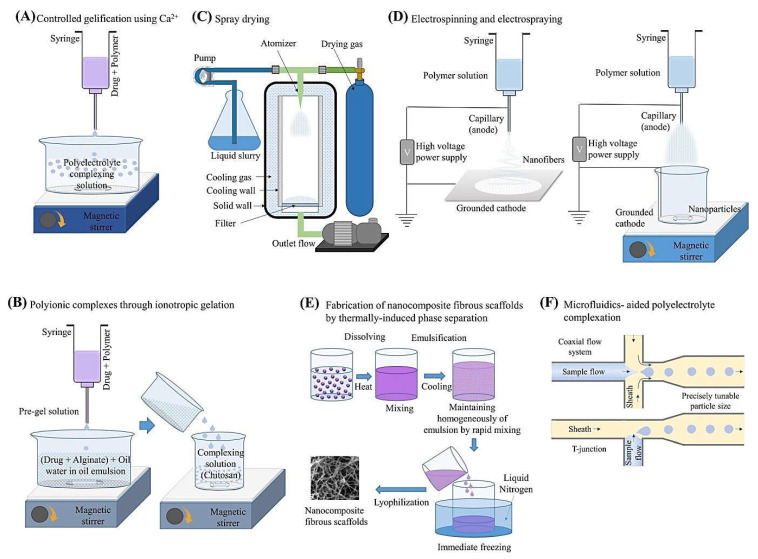
Schematic illustration of some important techniques employed for synthesizing alginate-based nanomaterials. Adapted from Ref. [13] with permission. Copyright 2019 Elsevier.

Alginates are natural polyanionic polysaccharides endowed with mucoadhesive property, biocompatibility, biodegradability, hydrophilicity, cost-effectiveness, non-toxicity, sol-gel transition features, and chemical versatility attributes and are produced by marine brown algae and bacteria [1,2,3,4,5]. In this context, alginate-based nanomaterials are gaining increasing attention particularly for drug/gene delivery, cancer therapy, and tissue engineering [6,7,8]. Alginates in combination with biomaterials offer attractive (nano)platforms in various forms such as hydrogels, nanogels, nanoparticles (NPs), magnetic (nano)systems, graphene oxide-based structures, microparticles, etc., for targeted and controlled delivery of therapeutic agents against cancers/tumors or malignancies (Table 1) [9,10]. These materials have shown excellent biomedical potential after enrichment via suitable functionalization [11,12]. Several preparative techniques have been introduced for synthesizing alginate-based nanomaterials, including controlled gelification using Ca^2+^ ions, generation of polyionic complexes via ionotropic gelation by intermolecular interactions, spray dying, self-assembly techniques, electrospinning/electro-spraying, thermally-induced phase separation, and microfluidic-aided polyelectrolyte complexation (Figure 1) [13]; some important challenges regarding the purity of industrially synthesized alginates along with their potential accumulation in body and biocompatibility issues are still persistent [13,14,15,16,17]. Their physicochemical properties can be affected by the extraction/processing and deployed synthesis procedures [6,18].

Due to the nanoporous nature of alginate gels, they are ideally suited for rapid diffusion of small molecules via the gel formation. The suitability of alginate gels is additionally felt when the kinetics of drug release can be adjusted by forming a primary or secondary bond between the drug and alginate [19]. Alginate hydrogels have been envisioned as suitable matrices for immobilization of nanomaterials, responsive polymers, and proteins, leading to a variety of stimuli-responsive nanosystems for cancer therapy and diagnosis [20]. In addition, alginate can be utilized for functionalization of different nanostructures, specifically for cancer treatment purposes [21]. For instance, biocompatible sodium alginate was exploited for functionalization of nanodiamonds for targeted delivery of cisplatin; these nanosystems exhibited improved drug safety along with enhanced drug accumulation and retention time in tumor cells, causing sustained chemotherapeutic drug release for tumor therapy [21]. Alginate-based nanosystems exhibited controlled drug release, increased stability, enhanced drug-loading capacity, and reduced immunogenicity, which renders them attractive biomaterials for cancer therapy applications [18,22]. Herein, recent advancements pertaining to the cancer therapeutic applications of alginate-based micro- and nanosystems are deliberated, with a focus on important challenges and future perspectives; discussions mostly center around the targeted drug delivery, photothermal therapy, and chemodynamic/photodynamic therapy.

**Table 1:** Some selected alginate-based micro- and nanosystems with cancer therapeutic applications.

Alginate-Based Systems	Applications	Approaches	Refs.
Alginate and collagen-based injectable hydrogels	Anticancer and anti-metastatic effects	Photothermal therapy and immunotherapy	[23]
Sodium alginate conjugated plasmonic magnetic nanocomposites	Drug delivery and cancer therapy	Targeted delivery of paclitaxel against human hepatocellular carcinoma cells	[24]
Sodium alginate–polyvinyl alcohol–bovine serum albumin coated Fe_3_O_4_ nanomaterials	Anticancer drug delivery with pH-responsive behavior	Controlled and targeted release of anticancer drug (doxorubicin) against cancer cells	[25]
Alginate–polydopamine hydrogels	Anticancer drug delivery with pH-responsive behavior	Cancer chemotherapy with chemo selective approach; targeted delivery of bortezomib to cancer cells	[26]
Chitosan–alginate nanosystems	Drug delivery and cancer therapy	Targeted delivery of doxorubicin with controlled and sustained release behavior	[27]
Tin oxide–sodium alginate–polyethylene glycol–carvacrol nanocomposites	Cancer therapy (against esophagus cancer)	Increase the generation of reactive oxygen species; enhance the pro-apoptotic and reduce the antiapoptotic proteins	[28]
Sodium alginate/phosphate-stabilized amorphous calcium carbonate nanocarriers	Drug delivery and cancer therapy	Targeted delivery of anticancer drugs/agents (curcumin) with sustained release and concentration-dependent behavior	[29]
Sodium alginate hydrogels	To monitor and obstruct postoperative recurrence and metastasis (in situ)	Cancer immunotherapy	[30]
Sodium alginate-based micelles	Anticancer drug delivery	Prolonged and targeted delivery of curcumin with blood-compatibility and stability	[31]
Curcumin–casein–alginate–chitosan nanocomplexes	Cancer nutraceutical therapy	Oral nano-delivery of curcumin with improved pharmacokinetics (enhanced bioavailability and cancer therapeutic efficacy against Ehrlich carcinoma)	[32]
Chitosan–sodium alginate–polyethylene glycol–crocin nanocomposites	Cancer therapy	Inhibition of the esophageal cancer KYSE-150 cell growth by enhancing the production of reactive oxygen species, and apoptotic cell death	[33]
Alginate-coated caseinate NPs	Anticancer drug delivery	Targeted and controlled delivery of doxorubicin against Ehrlich carcinoma	[34]
Chitosan/sodium alginate functionalized graphene oxide-based nanocomposites	Anticancer drug delivery	Targeted delivery of doxorubicin with pH-dependent drug release behavior	[35]
Alginate/chitosan-based nanosystems	Drug delivery	Encapsulation of hydrophobic quercetin with enhanced sustained release	[36]
Sodium alginate and hydroxyapatite bi-coated iron oxide NPs	Anticancer drug delivery	pH responsive controlled release of anticancer poorly water-soluble drug molecules (curcumin and 6-gingerol)	[37]
Epidermal growth factor receptor conjugated fucoidan/alginates loaded hydrogels	Cancer therapy (colon cancer)	Targeted photodynamic therapy	[38]
Fe_3_O_4_/calcium phosphate/alginate core-shell-corona NPs	Targeted chemotherapy	Targeted drug delivery with high biocompatibility and suitable particle size, surface functionality, and drug loading/release behavior	[39]

## 2. Alginate-Based Micro- and Nanosystems for Cancer Therapeutics

### 2.1. Targeted Anticancer Drug Delivery

Alginates are harnessed for encapsulating therapeutic agents with anticancer effects, providing stable and biocompatible nanosystems with improved targeting properties, low toxicity, and high efficiency. Alginate-based nanosystems have exhibited promising properties for encapsulating hydrophobic bioactive compounds with favorable safety profiles and no acute systemic toxicity (in vivo) along with enhanced protective activity for drugs. For instance, alginate nanogels were deployed for encapsulating *Artemisia ciniformis* extract with anti-proliferative activity and apoptotic effects on AGS gastric cancer cells [40]. Accordingly, these nanosystems exhibited greater potentials for inducing apoptosis and inhibiting cell proliferation when compared with free extract; the apoptosis induction occurred in a time-, and dose-dependent manner [40]. Besides, biocompatible alginate-based nanohybrids with stimulus responsive release (pH-sensitive behavior) and high encapsulation efficiency (~80.8 ± 10.6%) have been fabricated for sustained anticancer drug (doxorubicin) delivery [41]. These nanohybrids could be effectively internalized by osteosarcoma cell lines, exhibiting higher cytotoxicity to cancer cells than the free doxorubicin [41]. Tawfik et al. [42] introduced naturally altered nonionic alginate-based polymers as functionalizing agents for up-conversion of NPs. The designed nanosystems with high stability, biocompatibility, and luminescence intensity displayed alluring potentials for near-infrared imaging and anticancer drug delivery. Doxorubicin was successfully released in a highly controlled and selective pH-responsive manner through folate receptor-mediated endocytosis, thus providing nanosystems with efficient inhibitory effects against the growth of cancer cells [42].

One of the important challenges in multimodal cancer therapy is systemic dose-limiting toxicity, restricting its clinical applications. In one study, multi-responsive nanosystems were constructed from alginate hydrogels co-loaded with cisplatin and gold NPs for multimodal cancer therapy encompassing chemotherapy, radiotherapy, and photothermal therapy [43]. These nanosystems exhibited enhanced targeted drug delivery, resulting in excellent inhibition of tumor growth. In addition, the tumor treated with these nanosystems demonstrated an enhanced heating rate upon 532 nm laser irradiation, showing their suitable photothermal conversion potential. During the 90-day follow-up period, extensive tumor regression with no indication of relapse could be detected, with these alginate-based nanosystems for local synergistic cancer nanotherapy/tumor regression [43]. Liao et al. [44] introduced core-shell nanostructures constructed from inorganic Fe_3_O_4_ nanomaterials (the core), alginate (the shell), and cell-targeting ligands (D-galactosamine) decorated on the outer surface via the combined pre-gel and co-precipitation technique. These nanosystems with enhanced cellular uptake exhibited excellent hyperthermic efficacy in human hepatocellular carcinoma cell lines (HepG2), offering drug delivery systems for targeted cancer therapy (in vivo) [44]. To improve the anticancer drug delivery of nanosystems, natural peptide protamine sulfate and sodium alginate were applied for surface modification of graphene oxide-based platforms through layer-by-layer self-assembly technique [45]. The nanocomposites loaded with doxorubicin exhibited excellent pH-sensitive drug release profile with improved dispersibility and stability under physiological pH, thus revealing promising characteristics for targeted cancer therapy [45]. In addition, alginate hydrogels loaded with bevacizumab were fabricated for the delivery of anti-vascular endothelial growth factor to solid tumors at high concentrations, and to overcome the local therapeutic dose maintenance at targeted tumor [46]. In tumor sites, anti-angiogenic performance (~50%) was efficiently enhanced, and tumor size regression was remarkably obtained [46].

**Figure 2:**
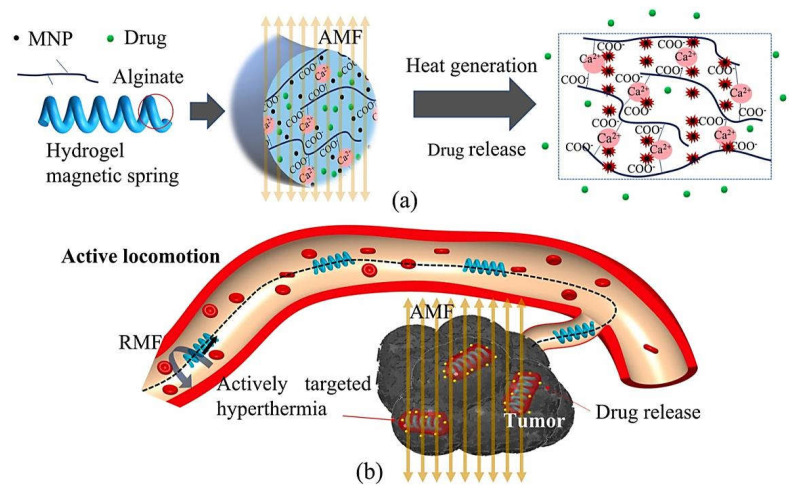
(**a**) Alginate hydrogel-based magnetic springs with active targeted hyperthermia and (**b**) drug release properties for cancer/tumor nanotherapy. AMF: alternating magnetic field; RMF: rotating magnetic field. Adapted from Ref. [49] with permission. Copyright 2022 Elsevier (CC BY 4.0).

Magnetic nanomaterials together with alginate have been applied for designing novel drug delivery (nano)systems for targeted cancer therapy [11]. For instance, curcumin-loaded hybrid magnetic alginate/Fe_3_O_4_ nanomaterials with pH-responsive behavior were constructed and complexed by bovine serum albumin and poly((3-acrylamidopropyl)trimethylammonium chloride) to produce novel drug delivery nanosystems for cancer therapy [47]. As a result, the curcumin anticancer drug was highly stabilized and its antitumor activity was enhanced; the loading efficiency of curcumin being ~95% [47]. Song et al. [48] designed curcumin-loaded magnetic alginate/chitosan nanomaterials with improved uptake efficiency, controllable/sustained release, high bioavailability, and cytotoxic effects towards MDA-MB-231 (Human Caucasian breast adenocarcinoma cells). These nanosystems exhibited suitable targeted delivery of curcumin with the help of magnetic field to reveal significant cytotoxicity to cancerous cells [48]. In addition, Zheng et al. [49] created calcium alginate hydrogel-based magnetic springs comprising magnetically aligned Fe_2_O_3_ magnetic nanomaterials to make them responsive towards magnetic fields, offering promising nanosystems for magnetic hyperthermia (Figure 2). This heat could produce thermal stresses, causing the spring’s mechanical distortion (shrinkage) that enhanced the drug release (~35% higher than that of physiological temperature) for active targeted heating and drug release [49].

**Figure 3:**
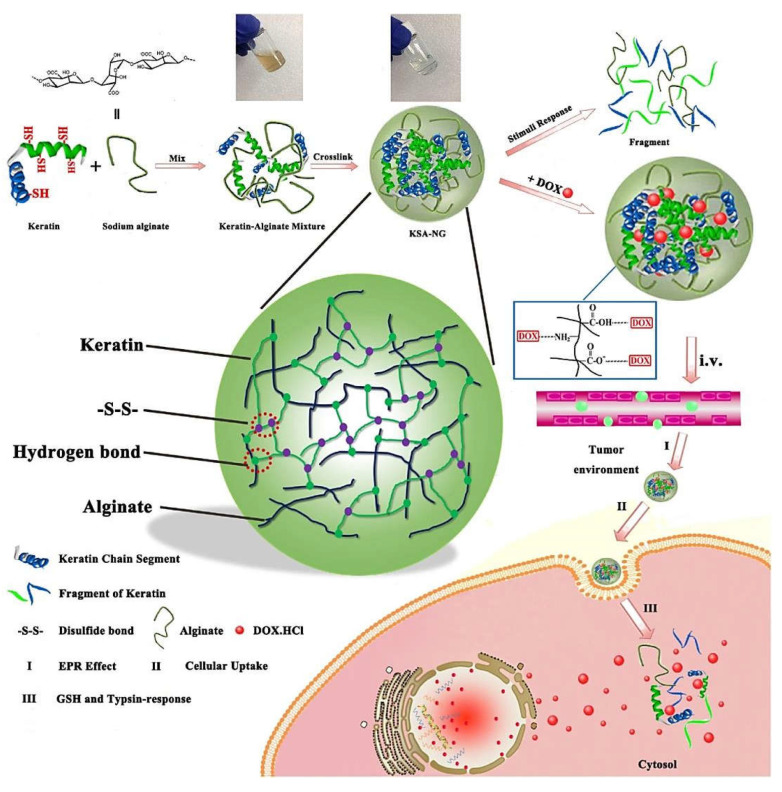
The preparative process for alginate–keratin composite nanogels and related mechanism of action (**I**–**III**), with bio-responsive behavior and improved drug loading efficiency for cancer nanotherapy. DOX: doxorubicin; EPR: enhanced permeability and retention; i.v.: intravenous; GSH: glutathione; KSA-NG: keratin-alginate nanogels. Adapted from Ref. [51] with permission. Copyright 2017 Elsevier.

Hydrogel-based (nano)systems have shown great potential for targeted anticancer drug delivery with high flexibility and controlled release behavior. Cao et al. [50] fabricated a hydrogel-based delivery system from poly(*N*-isopropylacrylamide), alginate, and graphene oxide–Fe_3_O_4_ nanomaterials, with near-infrared light-, magneto-, and pH-responsive drug release behaviors. The incorporation of alginate into hydrogels could improve the gelation and mechanical properties along with suitable pH-responsive function. The in vitro cytotoxicity evaluations illustrated that these nanosystems could effectively obstruct the cancerous cells, showing multi-responsive delivery for cancer therapy [50]. Furthermore, dual-stimuli responsive nanogels have been constructed from human hair keratin and alginate via cross-linking technique (Figure 3) [51]. Accordingly, keratin provided the cross-linking structure and bio-responsive capabilities, and alginate improved the stability and drug loading capacity of these nanogels. These nanogels (~120 nm) acted as vectors for targeted delivery of doxorubicin with significant drug loading efficiency (~52.9%) and dual-stimuli responsive behavior to glutathione and trypsin. Doxorubicin-loaded nanogels were successfully internalized in 4T1 and B16 cells (in vitro), with rapid drug release into cells under intracellular glutathione and trypsin levels, offering suitable nanosystems with anti-tumor effects and lower side effects compared to free anticancer drugs [51]. Similarly, smart nanogels (~432 nm) with high drug loading capacity (~65.2%) were designed using keratin (the multifunctional cross-linker) and sodium alginate [52]. These nanogels were capable of stimulating drug release upon both the pH and reductive (glutathione) environment while remaining stable under physiological conditions (pH 7.4 plus glutathione, 10 μM), showing efficient growth inhibition of HepG2 cancer cells; the loaded drug (~57%) was released at the simulated tumor intracellular microenvironment [52].

**Figure 4:**
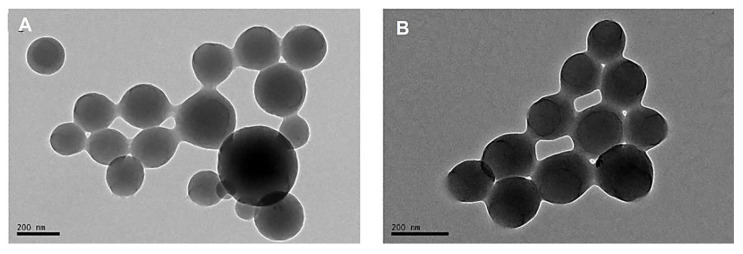
(**A**) Transmission electron microscopy (TEM) images of doxorubicin/alginate nanogels and (**B**) doxorubicin/collagenase@alginate nanogels. Adapted from Ref. [54] with permission. Copyright 2018 Elsevier.

Self-assembled core/shell NPs have been fabricated from water-soluble alginate substituted by hydrophobic phytosterols [53]. The conjugation of folate (the cancer-cell-specific ligand) to the phytosterol-alginate nanosystems was performed for targeted delivery of doxorubicin to folate-receptor-overexpressing cancer cells, providing suitable nanocarriers for targeting cancer cells overexpressing folate receptors and avoiding cytotoxicity to normal tissues [53]. Besides, alginic acid was co-polymerized with an acid-labile monomer to obtain the acid-degradable nanogels (hybrid pH-sensitive alginate nanogels), which was then immobilized with collagenase to form the surface-functionalized alginate nanogels (Figure 4) [54]. In this study, collagenase was introduced for enhancing the diffusion capability of nanogels in tumor parenchyma based on the hydrolytic activity to tumor extracellular matrix. Accordingly, the immobilization of collagenase highly improved the penetration of nanogels in tumor sites, causing higher drug concentration and remarkable antitumor effects [54].

### 2.2. Chemodynamic and Photodynamic Therapy

**Figure 5:**
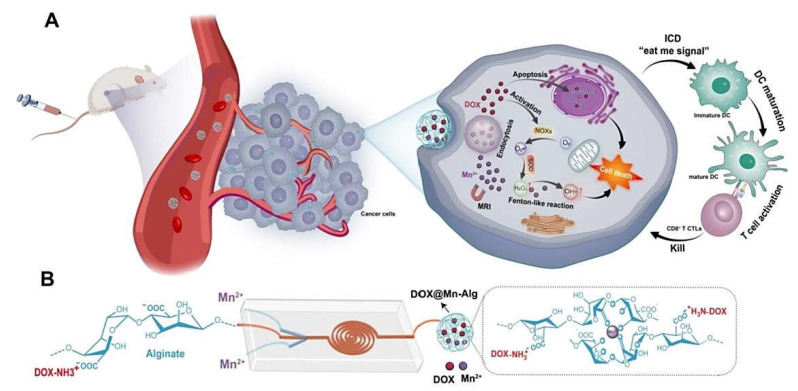
(**A**,**B**) Manganese–alginate nanogels prepared by microfluidic synthesis technique with self-supplying H_2_O_2_ and Mn^2+^-chelating capabilities for synergistic chemo/chemodynamic therapy and enhancing anticancer immunity. Mn: Manganese; Alg: alginate; DOX: doxorubicin; DC: dendritic cell; ICD: immunogenic cell death. Adapted from Ref. [55] with permission. Copyright 2022 Elsevier.

Chemodynamic therapy is based on the conversion of endogenous H_2_O_2_ into highly toxic hydroxyl radicals (^⋅^OH), causing the elimination of cancer cells [55]; in other words, it destroys cancer cells via the conversion of H_2_O_2_ or O_2_ into reactive oxygen species (ROS). However, the slow release of catalyst ions and deficient levels of H_2_O_2_ along with the limited tumor tissue penetration of the light source can severely restrict the performance of this tactic. To overcome these challenges, doxorubicin-loaded manganese–alginate nanogels were designed with self-supplying H_2_O_2_ potentials via the application of a microfluidic chip (Figure 5) [55]. These nanogels could rapidly release the anticancer drug in a pH-responsive behavior after internalization into tumor cells, revealing synergetic antitumor activities with no noticeable systemic toxicity. They also illustrated excellent potentials for magnetic resonance imaging cancer diagnostic applications. This chemodynamic therapy could stimulate the dendritic cell maturation and enhance the tumor infiltration of CD8^+^ T cells. The application of these nanogels with Mn^2+^-chelating and H_2_O_2_ self-supplying capabilities can be considered an attractive strategy for synergetic chemodynamic therapy with potentials for cancer immunotherapy [55].

Photodynamic therapy has shown some advantages of non-invasiveness and low toxicity, and can be applied for the elimination of cancer cells via the formation of cytotoxic ^1^O_2_ by irradiating photosensitizers with specific wavelengths of light [56]. However, one of the major challenges is that the excitation light of mostly deployed photosensitizers is in the UV/vis light region, thus limiting the application of this tactic mostly for epidermal or superficial tissue tumors [56]. In one study, the photosensitizer chlorin e6 and doxorubicin were adsorbed onto the magnetic mesoporous silica NPs [57]. After that, biocompatible alginate/chitosan polyelectrolyte multilayers were assembled on these NPs to generate a pH-responsive drug delivery system for cancer therapy, producing more singlet oxygen in cancerous cells after laser illumination. Accordingly, highly enhanced cell apoptosis (in vitro) could be attained by these nanosystems (~280 nm). The combinational therapy using photodynamic and chemotherapy approaches could synergistically have efficient antitumor effects (in vivo) [57]. Similarly, the conjugation of pheophorbide A (a hydrophobic photosensitizer) was performed through a redox-sensitive disulfide linkage to alginate to construct redox- and light-responsive alginate-based nanosystems as effective drug nanocarriers [58]. Doxorubicin was loaded next for targeted combinational tumor therapy purposes. These nanosystems (~210 nm), with efficient cellular uptake by B16 tumor cells (murine melanoma) and intracellular ROS formation along with the enhanced uptake of doxorubicin and pheophorbide A, could be applied for tumor chemotherapy and photodynamic therapy, resulting in successful growth inhibition of tumors [58].

### 2.3. Photothermal Therapy

**Figure 6:**
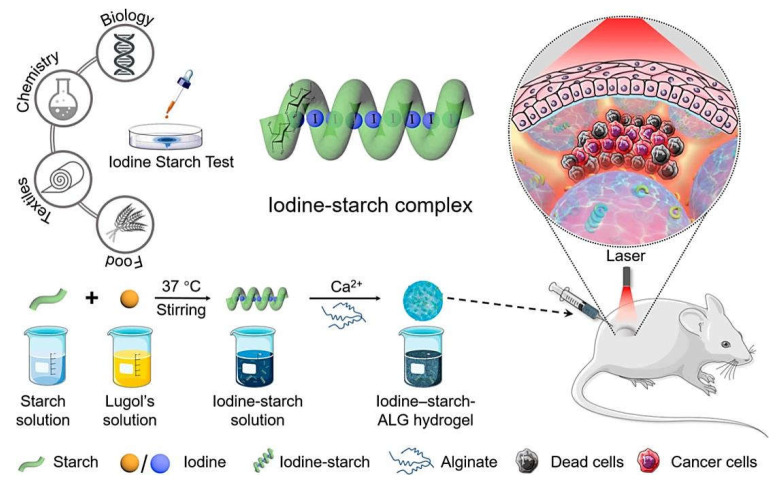
The preparative process for biocompatible iodine–starch–alginate hydrogels through ionic cross-linking of Ca^2+^ and alginate for targeted tumor photothermal therapy. Adapted from Ref. [59] with permission. Copyright 2019 American Chemical Society.

Photothermal therapy has been applied for tumor/cancer therapy in view of the attractive benefits of minimal invasiveness and high efficiency; however, clinical applications of photothermal therapy agents have been restricted because of their safety concerns. For instance, biocompatible iodine–starch–alginate hydrogels were constructed for tumor photothermal therapy (in vitro/in vivo) (Figure 6) [59]; these hydrogels exhibited strong absorption at 808 nm, indicating their potentials in photothermal therapy. Notably, the iodine–starch complex contained dark blue iodine–amylose complex (with maximum absorption at 650 nm) and reddish-purple iodine–amylopectin complex (with maximum absorption at 540 nm). These high-performance hydrogels with good biocompatibility and degradability exhibited excellent photothermal heating potentials based on iodine–starch chromophore; the alginate–Ca^2+^ hydrogel played a crucial role in refining the chemical stability of iodine–starch by reducing the interactions between iodine and enclosing reductive molecules [59]. In addition, alginate-based nanogels co-loaded with Au nanomaterials were designed for targeted delivery of cisplatin (chemotherapy) combined with photothermal therapy (under 532 nm laser irradiation) against CT26 colorectal tumors [60]. This chemo-photothermal therapy with synergistic effects exhibited significant tumor suppression (~95%), providing attractive alginate-based nanoplatforms for inhibiting tumor growth as well as the removal the microscopic residual tumor [60].

Combination therapy has been one of the most important strategies in the field of cancer therapy, especially against tumor re-occurrence and metastasis. Graphene oxide nanosheets synthesized by solvothermal reaction were enriched by magnetic iron oxide NPs, and then functionalized with chitosan and sodium alginate via non-covalent layer-by-layer self-assembly technique to generate nanocomposites for combinational targeted anticancer drug delivery and photothermal therapy [61]. These nanocomposites with superparamagnetic property, enhanced stability, pH-responsive release behavior, high drug loading capacity, and reduced agglomeration in biological solutions could be loaded with doxorubicin. Accordingly, excellent magnetically targeted cellular uptake properties and photothermal effects were displayed by these nanocomposites, thus illustrating the promising role of alginate in the assembly of smart targeted cancer therapy nanosystems [61]. Sheng et al. [62] introduced chemo-photothermal synergistic therapy nanosystems constructed from graphene oxide and hydrogels of oxidized alginate/carboxymethyl chitosan for targeted delivery of methotrexate and naringin against osteosarcoma. In addition to drug delivery, these nanosystems were applied for photothermal therapy, producing hyperthermia under near-infrared irradiation for ablation of the osteosarcoma cells. The cumulative release of methotrexate and naringin was ~91.09% and ~85.69%, respectively, at pH 5.0 with near-infrared irradiation [62]. Besides, injectable collagen/alginate hydrogels were fabricated for combinational antitumor therapy using photothermal drug methylene blue and immunological agent imiquimod (R837) [63], ensued biocompatible nanosystems were deployed for localized delivery and sustained/controlled release of therapeutic agents, offering excellent combinational photothermal and immuno tumor therapy efficiency [63].

## 3. Challenges and Future Perspectives

Alginate-based micro- and nanosystems exhibit high drug loading capacity and controlled-release behavior, and can be simply modified/functionalized through chemical modification or easy preparative strategies [64,65]. Overall, a variety of targeted drug delivery systems have been introduced using alginates, with passive targeting, active targeting, and stimuli-responsive release mechanisms. The enhanced permeability and retention effect (recognized as passive targeting) permits the nanoscale carriers to be distributed explicitly in the tumor at high concentrations and taken up by cells more efficiently. On the other hand, by conjugating to the ligand-receptor, antigen-antibody, and other types of molecular recognition means onto drug delivery systems, active targeting by alginate-based systems can be achieved. Stimuli-responsive drug delivery systems typically involve a phase transition in response to the micro-environmental alterations of cancerous cells, including pH, temperature, specific ions, etc. In addition, several stimuli-responsive alginate-based drug delivery systems have been designed in which the drug release could be stimulated by external non-invasive signals such as light, heat, ultrasound, and magnetic fields [18,64]. Among these strategies, active targeting and stimuli-responsive release behaviors have been mostly focused by researchers to prevent or reduce the off-target effects, undesirable biodistribution, and low therapeutic efficacy [66]. In this context, dense extracellular matrix as one of the crucial barriers obstructs the penetration of drugs into tumor parenchyma, which can affect the therapeutic efficacy [54].

Among introduced polymers, alginates with an abundance of free hydroxyl and carboxyl groups distributed along the polymer chain backbone can be simply modified with specific functional groups or ligands to acquire cancer targeting functionality [64]. Alginate-based systems with biodegradability, biocompatibility, and non-toxicity advantages can be employed for targeted and site-specific anticancer drug delivery along with photothermal, chemodynamic, and photodynamic cancer therapy. However, several crucial aspects regarding their stability, possible aggregation, mechanism of action, bio-clearance, and clinical translational studies are still lingering challenges. It appears that with advancements in chemical and biomedical engineering, novel micro- and nanosystems with improved properties and functionality can be designed for cancer theranostic purposes, including both therapeutics and diagnostics/imaging [9]. In this context, multifunctional anticancer-drug nanocarriers ought to be further developed via the incorporation of functional nanomaterials such as near-infrared-responsive gold nanorods and superparamagnetic iron oxide NPs functioning as magnetic resonance imaging contrast agents [13,67]. In one study, multifunctional carriers were designed using alginate, gold nanorods, and superparamagnetic iron oxide NPs for targeted and controlled delivery of doxorubicin into the external environment upon irradiation with near-infrared laser and imaging. These nanosystems can be specifically monitored with the magnetic resonance T_2_ imaging mode [67]. Another important challenge is the targeted delivery of drugs with hydrophobicity. Since most of the anticancer drugs have shown low solubility in water, thus their clinical applications would be restricted unless altered by suitable modification processes deployed for enhancing the solubility as exemplified by the development of alginate-based magnetic nanocarriers for the targeted delivery of hydrophobic drug molecules such as doxorubicin and paclitaxel [68]. The alginate shell around the magnetic core could enhance the stability and biocompatibility of loaded drugs, offering nanosystems with low toxicity and faster release behavior in acidic medium than in the neutral medium [68].

## 4. Conclusions and Future Outlooks

Alginate-based micro- and nanosystems with unique properties and multifunctionality have been explored for the targeted cancer therapy and drug delivery. The application of alginate for encapsulation of anticancer drugs not only can lead to controlled and sustained drug release but also highly improve the effectiveness of anticancer drugs against cancers. In addition, the presence of alginate can improve the stability of nanosystems in the acidic environments of biological fluids. However, since limited research has been performed in vivo, it is not possible to have a comprehensive analysis on the clinical efficacy of these drug delivery systems. Therefore, extensive and comprehensive clinical translational and in vivo studies are vital in the fabrication of advanced drug delivery nanosystems. Apparently, it is the need of the hour to further investigate the biosafety/toxicity and biocompatibility of these materials as well as the optimal production techniques based on green chemistry principles; crucial parameters including solubility, reactivity, and characterization would help determine the derivatization and design strategies for the alginates.

Hyperthermia strategy for cancer therapy ought to be further explored, especially pertaining to the application of novel photothermal and magnetic fluid agents for selectively targeting tumor sites and efficiently elevating temperature while preserving biocompatibility attributes. Alginate biopolymers can be applied for improving the poor dissolution rate and oral bioavailability of anticancer drugs. Thus, future explorations ought to be conducted to unearth novel alginate-based nanosystems for targeted cancer therapy using advanced oral drug delivery tactics with the added benefits of non-invasiveness, patient compliance, and convenience of drug administration. However, several challenging issues regarding the mucosal permeability, stability in the gastrointestinal tract environment, and drug solubility still need to be systematically evaluated and addressed. To minimize the undesirable effects and improve the efficiency/targeting properties, natural biopolymers-based pharmaceutical delivery strategies can be adapted as one of the alternative methods in drug delivery instead of conventional techniques. This category will be of interest to the pharmaceutical industry when all-inclusive clinical studies need to be conducted for them with the acceptable economic justification for the industry. Optimizing the production methods and their reproducibility, along with the use of renewable/sustainable and low-cost materials, would go a long way for acceptance of such strategies.

## Data Availability

Not applicable.
